# Relaunch of the official community health worker programme in Mozambique: is there a sustainable basis for iCCM policy?

**DOI:** 10.1093/heapol/czv036

**Published:** 2015-10-29

**Authors:** Baltazar GM Chilundo, Julie L Cliff, Alda RE Mariano, Daniela C Rodríguez, Asha George

**Affiliations:** ^1^Faculty of Medicine, Eduardo Mondlane University, Salvador Allende Ave., Maputo, Mozambique and; ^2^Department of International Health, Johns Hopkins Bloomberg School of Public Health, Baltimore, MD, 21205 USA

**Keywords:** Community health workers, donors, iCCM policy, Mozambique, NGO coordination, sustainability

## Abstract

**Background:** In Mozambique, integrated community case management (iCCM) of diarrhoea, malaria and pneumonia is embedded in the national community health worker (CHW) programme, mainstreaming it into government policy and service delivery. Since its inception in 1978, the CHW programme has functioned unevenly, was suspended in 1989, but relaunched in 2010. To assess the long-term success of iCCM in Mozambique, this article addresses whether the current CHW programme exhibits characteristics that facilitate or impede its sustainability.

**Methodology:** We undertook a qualitative case study based on document review (*n* = 54) and key informant interviews (*n* = 21) with respondents from the Ministry of Health (MOH), multilateral and bilateral agencies and non-governmental organizations (NGOs) in Maputo in 2012. Interviews were mostly undertaken in Portuguese and all were coded using NVivo. A sustainability framework guided thematic analysis according to nine domains: strategic planning, organizational capacity, programme adaptation, programme monitoring and evaluation, communications, funding stability, political support, partnerships and public health impact.

**Results:** Government commitment was high, with the MOH leading a consultative process in Maputo and facilitating successful technical coordination. The MOH made strategic decisions to pay CHWs, authorize their prescribing abilities, foster guidance development, support operational planning and incorporate previously excluded ‘old’ CHWs. Nonetheless, policy negotiations excluded certain key actors and uncertainty remains about CHW integration into the civil service and their long-term retention. In addition, reliance on NGOs and donor funding has led to geographic distortions in scaling up, alongside challenges in harmonization. Finally, dependence on external funding, when both external and government funding are declining, may hamper sustainability.

**Conclusions:** Our analysis represents a nuanced assessment of the various domains that influence CHW programme sustainability, highlighting strategic areas such as CHW payment and programme financing. These organizational and contextual determinants of sustainability are central to CHW programme strengthening and iCCM policy support.

Key Messages

- In Mozambique, integrated community case management policy is embedded in the revived national community health worker programme.
- Embedding the programme in the Ministry of Health structures not only facilitated sustainability but also made it vulnerable to health system constraints, such as weak supervision and poor logistics.
- Although the Ministry of Health led the development and implementation processes, a reliance on donor funding and a donor-led agenda threatened sustainability in the long term.
- To ensure sustainability, government commitment needs to extend to financing through the state budget and incorporating community health workers in the civil service.

## Introduction

In 2002, Mozambique signed up to the millennium development goals (MDGs), committing to reduce the under-five mortality rate by two-thirds between 1990 and 2015 (Ministério da Saúde, 2008). By 2012, the rate fell to 89.7/1000 from 232.6/1000 in 1990, a 61% reduction (United Nations, 2013). This fall overlapped with a sustained post-war economic recovery and the provision of effective health interventions, such as immunization and the integrated management of childhood illnesses (IMCI), through an expanded health care network (Governo de Moçambique, 2010). Despite these positive gains, access to health services remained low, with around 56% of the population taking over an hour to reach the nearest health facility (Ministério da Saúde, 2007a). The Ministry of Health (MOH) recognized that further reductions in child mortality required more emphasis at the community level. In 2004, the MOH approved a strategy for community involvement, which included the training and placement of community health workers (CHWs) (Ministério da Saúde, 2004).

The community strategy was a continuation of a longstanding national commitment. As part of the historic decision to embark on primary health care, Mozambique established a programme of CHWs called Agentes Polivalentes Elementares (APEs) in 1978 (Lindelow *et al.*, 2004). APEs were trained to carry out health promotion and disease prevention activities, provide first aid, treat common diseases (including malaria with chloroquine and dehydration from diarrhoea with oral rehydration) and refer patients to the nearest health facility (Ministério da Saúde, 1977). By the early 1980s, the APE programme ran into difficulties, partly attributed to a war which lasted from 1977 to 1992. Other challenges included: (1) the community perceived APEs as the most peripheral service delivery point of the national health system (NHS) and not as an integral part of the community they served; (2) a widespread perception of APEs as providers of curative services, in conflict with their training, which emphasized preventive services; (3) APEs wanted to be part of the NHS; (4) weak supportive supervision and monitoring; and (5) an outdated curriculum. Given these problems, the MOH suspended the APE programme in 1989 (Ali *et al.*, 1994). Nonetheless, many APEs continued working, often supported by non-governmental organization (NGO). The MOH also continued to supply APEs with medicines through a kit system adopted in the 1980s. Sporadic attempts were made by the government to revive the APE programme, with revised training and support manuals produced in 1992 and 1993.

Following approval of the new community strategy in 2004, community IMCI gained traction with the launch of the national policy of neonatal and infant health in 2006. The policy proposed mobile brigades, village health days and treatment of common childhood illnesses at the community level to address the lack of access to health facilities (Ministério da Saúde, 2006a). Subsequently, the MOH led a range of actions to adapt and implement community IMCI with strong support from bilateral and multilateral cooperation partners (Notably Canadian International Development Agency (CIDA), Swiss Development Cooperation, United Nations Children’s Fund (UNICEF), United States of America Agency for International Development (USAID), the World Bank and World Health Organization (WHO)) and some Non Government Organizations (NGOs) (e.g. Save the Children, Malaria Consortium and World Vision).

In 2010, actions to strengthen community IMCI culminated with the launching of the APE Revitalization Programme (Ministério da Saúde, 2010a,b). Important tools for operationalizing the programme were developed, and training began a year later (Ministério da Saúde, 2012b). The APE programme includes antibiotics for pneumonia, oral rehydration salts and zinc for diarrhoea and artemether/lumefantrine for malaria, i.e. integrated community case management (iCCM). It aims to extend community access to health care by 20% and deploy 6343 APEs by 2015/16 (Ministério da Saúde, 2010b). As of December 2013, there were 2270 APEs trained across 117 districts covering 12% of the population (Ministério da Saúde, 2014b).

Given the earlier decline of APEs, a key question is whether the current revitalization rests on a more sustainable foundation. To assess the long-term likelihood of success of iCCM policy in Mozambique, this article addresses the following research question ‘Does the current revitalization of the APE programme, which encapsulates iCCM, exhibit characteristics that facilitate or impede its sustainability?’

A simple definition of sustainability is the ‘capability of being maintained at a certain rate or level’ (Gruen *et al.*, 2008). For health programmes, sustainability can be regarded as ‘the ability … to function effectively, for the foreseeable future, with high treatment coverage, integrated into available health care services, with strong community ownership, using resources mobilized by the community and government’ (APFO and WHO, 2004). Sustainability therefore requires attention to broader organizational and systems dynamics (Sarriot *et al.*, 2004) and as such is also defined as ‘the long term ability of an organizational system to mobilize and allocate sufficient and appropriate resources (manpower, technology, information and finance) for activities that meet individual or public health needs and demands’ (Olsen, 1998). Accordingly, several reviews stress its dynamic nature, fuelled by interactions between stakeholders, institutions and beneficiaries and ensuring continuation through adaptations to broader environments (Shediac-Rizkallah and Bone, 1998; Gruen *et al.*, 2008; Wiltsey Stirman *et al.*, 2012).

A conceptual framework proposed by Schell *et al.* (2013) categorizes the determinants that support long-term programme sustainability in public health. The framework lists nine domains, in two inter-related loci: (1) internal factors such as strategic planning, organizational capacity, programme adaptation, programme monitoring and evaluation, and communications; and (2) external factors such as funding stability, political support, partnerships and public health impact. We adapted this nine-domain framework to examine the basis of sustainability of the APE programme and, by extension, of iCCM policy in Mozambique.

## Methods

We undertook a qualitative retrospective case study (Yin, 2009), as part of a larger policy analysis of six sub-Saharan African countries that used a multiple case design with theoretical replication (Bennett *et al.* 2014). Documents were collected and interviews conducted in 2012, in Maputo, the capital city, where most of the key stakeholders in the development and implementation of the APE programme/iCCM policy are based.

The document review analysed published and unpublished information from a variety of sources (Table 1) to contextually ground the study and adapt the interview guide to the policy environment of the APE programme and iCCM policy in Mozambique. Subsequently, during interviews, respondents were asked to provide relevant policy and programme documents, such as strategic frameworks, action plans and progress reports.

**Table 1.** Number and types of documents reviewed

Document type	National	International	Addressing iCCM	Addressing other aspects
Research articles	1	5	4	2
Reports	20	3	9	14
Government Reports	33		13	20
Books		2		2
Total	54	10	26	38

After receiving approval from both the National Bioethics Committee and the MOH, a potential list of respondents was developed based on the document review, prior experience related to the study topic and through snowball sampling once interviews started. Respondents were invited to participate in the study through telephone, email and by post. Of 40 potential participants, 14 did not respond despite two attempts to reach them and five either refused or were not able to do the interview, leaving 21 respondents who accepted and completed an interview (Table 2). Almost all interviews were face-to-face and took place at the respondent’s preferred site in Portuguese (only two were conducted in English, following the respondent’s choice). Most interviews lasted ∼45–60 min, but a few were shorter given the time pressures of busy managers. With the permission of the participant, almost all interviews were digitally recorded and transcribed. Interviewers also took notes to enrich information from the recordings.

**Table 2.** Respondents approached and interviewed by type of organization

Respondent type	Number contacted	No response or not able to do	Number interviewed
Government	14	6	8
Multilateral organizations	7	2	5
Donors and bilateral organizations	8	7	1
International NGOs	8	3	5
Other actors	3	1	2
Total	40	19	21

## Analysis

A skeleton coding structure was developed by the JHSPH team in collaboration with national researchers following the concepts explored through the interviews. These concepts were informed by Walt and Gilson’s policy triangle (Walt and Gilson, 1994) and included consideration of policy content, policy processes, actors who were involved in (supporting or opposing) policy development and contextual issues such as human resources for health, financing and use of evidence. Thematic coding was systematically done using NVivo software.

## Results

iCCM policy in Mozambique is embedded in the national APE programme (Ministério da Saúde, 2010a,b) and is thus mainstreamed into government policy and service delivery. iCCM is therefore shaped by the strategic decisions made for the APE programme; gaining from its strengths, but also vulnerable to its weaknesses. Table 3 summarizes the facilitators of and barriers to APE sustainability in Mozambique, using the sustainability framework discussed earlier (Gruen *et al.*, 2008; Schell *et al.*, 2013). We first present the internal factors that are critical for sustainability (strategic planning, organizational capacity, programme adaptation, programme evaluation and communication) and then the external factors (funding stability, political support, partnerships and public health impact).

**Table 3.** Sustainability of iCCM in Mozambique: facilitators and barriers

Domains	Facilitators	Barriers
Internal factors		
Strategic planning: defines programme direction, goals and strategies	APE programme developed in a consultative manner across MOH departments and with partners National policies and guidelines reviewed to avoid mistakes made in the past, e.g. APE non-payment After consultative process with drug regulatory agencies, MOH exercised fiat regarding drug regulations allowing APEs to prescribe certain medicines and mainstreaming medicines into the NHS and APE kit	Poor coordination with MOH departments Ministry of Finance not included in consultations APEs have short-term contracts, low pay (not full-time salary) and no career path, causing potential retention problems APEs not integrated into the civil service, due to their educational level, despite precedence from other Ministries on how to incorporate community level agents into government structures
Organizational capacity: resources to manage the programme and its activities	Operational guidelines and tools developed APEs trained in standardized manner APEs equipped with necessary equipment and supplies to carry out their tasks NGOs with programme experience willing to support supervision and logistics	Weak supply chain, with frequent medicine stock-outs may demotivate APEs and the community Weak supportive supervision systems Dependence on NGOs/ partners and difficulties with harmonization may weaken government health systems and oversight
Programme adaptation: improvements to ensure effectiveness	Decision to upgrade old APEs and include them in the revitalized programme Slow and careful implementation enabled learning, problem solving and adaptation	Delays to retrain old APEs are mostly male presenting a challenge for home visits to and care of pregnant and post-partum women and newborns
Programme evaluation: monitoring and evaluation of processes and outcomes	Standardized registers and reporting documents approved Government and partners would like the APE programme to demonstrate impact	Disparate donor/partner/NGO requirements resulted in a multiplicity of monitoring and evaluation tools, weakening the information system Research agenda for iCCM/APE not elaborated/supported
Communication: with stakeholders, decision-makers, the public	Routine technical working group meetings held at the central level to ensure dissemination and coordination of APE/iCCM efforts	Lack of communication with district actors led to continued building of health posts for APEs, perhaps undermining preventive/promotive activities Poor communication with Ministry of Finance
External factors		
Funding stability: long-term planning and stable funding environment		Programme is entirely dependent on external donors for salaries, drugs, supplies, supervision, etc. Scale up is slow as government requires partners to pay for APEs comprehensively and not just for training Funding partners targeting specific provinces and districts, leaving others without support, leading to geo-discrepancy in service delivery and unequal distribution of APEs Weak and decreasing contribution of the state budget to the health sector Decrease of external support to the health sector, pending response from global fund audit recommendations
Political support: internal and external political environment	Strong national government commitment to community engagement and the APE programme iCCM included as top priority for achieving MDG4	
Partnerships: NGOs and communities	NGOs accountable to donors for progress on iCCM Community members value APEs	Community beneficiaries not mobilized in planning process
Public health impact: effect on population health attitudes, perceptions and behaviours	Potentially positive, but not yet measured	Research agenda for iCCM/APEs not yet elaborated or supported

*Source:* Adapted from Schell *et al.* (2013).

### Internal factors

#### Strategic planning

Strategic planning involves the establishment and facilitation of key relationships to make critical decisions regarding programme direction, goals and strategies. We review the policy and planning processes for the new APE programme, before discussing two key strategic issues; APE payment and inclusion in the civil service and authority to prescribe medicines.

#### Policy and planning processes

The planning process for the new APE programme, and hence iCCM, spanned 6 years, starting in 2004 and crystallizing in 2010 with the launching of the new programme. Inspired by the sector-wide approach, the MOH and its partners organized various expert technical working groups, a process similarly used to develop other national health policies and strategies.

Within the MOH, the National Directorate of Public Health, which included the Departments of Women’s and Child Health and Health Promotion, played the leading role. The National Malaria Control Programme was also active as its strategic plan included community malaria case management (Ministério da Saúde, 2006b, MOH-NMCP, 2009). There was also strong involvement of the National Directorate of Human Resources (particularly the Deputy Directorate of Training).

Despite this collaboration across various departments, some respondents reported ‘weak’ coordination. Unexpected considerations caused delays, e.g. the need to harmonize the definition of the APE profile with APE selection criteria and curriculum design.

“… there was not very good communication … partners had to apply a lot of pressure from the outside … the APE programme, asked the department of training to develop training materials [for APE programme] but it [the APE programme] had no idea that this was to ask much more than that, it was necessary to develop the APE profile, the curriculum and define where this programme is to be managed.”—NGO respondent [2]

The MOH facilitated broad consultations with external actors (NGOs, bilateral and multilateral organizations) in Maputo. Pressure from these partners was coupled with strong leadership from the MOH, with the Ministry successfully managing to move all its collaborators and partners in one single direction: ‘There were several working groups, but the leadership was certainly from the MOH’—NGO respondent [11].

The Ministry of Finance was a notable exception to this inclusive process at national level.

“The Ministry pledged with partners and donors that during the two years in which partners and donors would support the programme they would work with the Ministry of Finance to absorb these people, once again there was no involvement of the right people.”*—*NGO respondent [2]

In contrast to government technocrats and partner organizations, neither APEs themselves nor district or community level actors were extensively engaged or mobilized in the planning process.

#### APE payment and inclusion in civil service

In the hope of avoiding past problems, the MOH and partners initially proceeded with an extensive review of the earlier APE programme (Ministério da Saúde, 2010b). As the earlier programme did not provide stipends and serious problems of retention arose, in the new programme, all APEs receive a monthly stipend equivalent to US$40, which is less than a fulltime salary. However, motivation and retention remain concerns due to short-term contracts, low salaries and lack of an explicit career path.

Also despite agreeing to a stipend, the government does not directly pay APEs, because the public service law requires a minimum academic qualification of grade 10 (basic level) for Technical Assistant and Auxiliary employees (Assembleia da República, 2009), which excludes APEs as the APE programme only requires minimal literacy (ability to speak, read and write Portuguese satisfactorily) and basic arithmetic (Ministério da Saúde, 2010a). Nonetheless, respondents also pointed out that 

APEs could possibly be admitted under another category (the General Statute of the State Officials and Agents), which only requires Grade 7 level qualification (Assembleia da República, 2009). The MOH did consult other ministries to examine their experience of paying less educated workers, such as literacy instructors, agricultural extension workers or even community leaders.

“We know there are other areas like agriculture and education where they have found ways to hire Activists and Community Workers by having a contract and receiving subsidies from government.”*—*Multilateral organization respondent [5]

“… we limited ourselves saying that this would be done in the same way as the Ministry of Education did with Alphabetizers, the Ministry of Agriculture with Extensionists. … now we do not know how this will be solved, because there are laws that must be complied with, it is important to assure the APE subsidy from the state budget, otherwise the programme is not sustainable … ”—NGO respondent [2]

Until the government finds a long-term solution, partners have agreed to temporarily provide funds for APE salaries.

#### APE authority to dispense medicines

Difficulties also arose around the issue of drug prescription. iCCM policy requires that APEs prescribe amoxicillin and artemether/lumefantrine, which conflicts with the government norm regarding which drugs APEs can prescribe (Governo de Moçambique, 2007). This issue led to heated debate within the National Pharmacy and Therapeutics Board, where many members argued that APEs should not be allowed to prescribe a medicine above their level in the system (Comissão Técnica de Terapêutica e Farmácia, 2011). In addition, dispersible amoxicillin and zinc, used for iCCM, had yet to be introduced into the NHS and were considered too expensive to be included in the APE medicine kit (Kit C).

“… at the time the APE package was being developed, I remember that there was some resistance from within the ministry or internal discussions about zinc or dispersible amoxicillin because these products were not available in the health facility … this forced the ministry to include them in the public system in order to avoid creating discrimination … where a community worker would have products of better quality than the health facility.”*—*Multilateral organization respondent [5]

After several meetings, the Board finally allowed APEs to prescribe these medicines, but only due to ‘perceived pressure’ from the MOH.

“… What I know is that there was much debate. We … discussed it because these formulations [dispersible] are too costly and would make Kit C very expensive, but because the decision was taken already, we had to accept … ”—Government respondent [6]

#### Organizational capacity

To support the development of organizational capacity, the MOH produced operational guidelines and tools, including a guideline and curriculum for the APE course, training materials, key points for implementation, supervision and management guides, and monitoring and evaluation tools (Ministério da Saúde, 2010a,b, 2011a).

To support their motivation and performance, APEs are supplied with a bicycle, bags, waistcoat, lantern, tape-measure for arm circumference, a stop watch and a basic medicine kit (Ministério da Saúde, 2010a). However, the programme’s logistics system faced challenges which contributed to medicine stock-outs: (1) no standardized system for reporting logistics data or resupply, (2) limited APE ability to track data and store commodities properly at their homes, and (3) poor communication between APE coordinators and medical stores at both district and provincial levels (USAID Deliver Project, 2013).

Organizational capacity to support supervision, key for both quality and sustainability, is also weak. Ideally, APEs receive a monthly supervision visit from a health professional based in their referral health facility. Also, they receive supervision at the facility when they collect their kit and present their monthly report of activities (Ministério da Saúde, 2011b). In practice, as highlighted by many respondents and policy documents, supervision has been poor:

“Poor supervision and support, particularly at the most peripheral levels, conditions the quality of services provided to this important level of health care, which often represents the first and only contact for those who seek health care” (Ministério da Saúde, 2014a, p. 77)*.*

The government currently relies on the presence of NGOs with programme experience to support supervision and logistics. While positive, this reliance entails coordination to ensure harmonization and care to ensure that it does not displace strengthening of local health systems and government oversight.

“To me supervision is one of the technical issues that is priority. This has not been sorted out. Now I’m hearing WHO/CIDA coming in with funds for NGOs … we are trying to advocate one NGO for each province to do that work of supervising, and ensuring the monitoring is taking place whereby district authorities go out and do this job properly.”—Multilateral organization respondent [21]

#### Programme adaptation

Inevitably unplanned difficulties led to adaptation during implementation. For example, some provinces still had ‘old’ APEs providing health services with their previous medicine kit. With the shift to the new APEs, these old APEs were stopped from collecting their medicine kit, depriving their communities of their services. Meanwhile, the new APEs were still too few in number to compensate for the loss of the old APEs.

“Niassa … province had about 400 old APEs receiving monthly kits, but now the ministry is not giving the kit to the old APEs, so Niassa is losing, as now there are only 100 to 150 new APEs being trained. It is from here that a decision was made to upgrade old APEs and to make sure communities are not lagging without health services that they had before.”—Multilateral organization respondent [5]

Upgrading old APEs to ensure quality proved challenging, as they previously were trained using a diverse range of guidelines and curricula developed by many partners without clear guidance by the MOH. This is being taken into account by the MOH:

“we have done a rapid assessment and could realize that in the field there are many APEs trained differently, some with only 2 months, others with 3, 4 and 6 months … so will elaborate another assessment to know who are the people to be trained.”—Government respondent [3]

Most respondents (with some exceptions), were unanimous in emphasizing that, although the process of setting up the conditions for implementation had been lengthy, this was preferable to a faster implementation that could compromise. Slow and careful implementation had enabled learning, problem solving and adaptation to difficulties as they arose.

“Yes, by going slowly we go a further distance. … we are in this consensual process in order to train more and be able to reach all districts, the outreach population … I think even slowly we will get there … ”—Government respondent [3]

A key issue for quality and continuity is that, despite programme guidance prioritizing female candidates, only one out of five trained APEs is female. APEs are selected by their community, whose choice of young men has been respected. As well as pay, communities may perceive that APEs have an opportunity for advancement, which men are perceived to need more as traditional breadwinners. Young men also tend to have higher literacy than young women.

As one respondent stated:

“It’s a question of lack of opportunity and unemployment at the rural level, that may make men seek it out as a source of income … We want women because later when we will be introducing other aspects related to women, it will be easier.”—Research Organization respondent

This preponderance of male APEs may deter women from seeking care for their children, particularly newborns. Care after birth for the mother and newborn is in the hands of female relatives, and traditional midwives and men are excluded. The strength of this exclusion is illustrated by a respondent:

“The Ministry of Health is afraid; it thinks that an APE cannot deal with a newborn, first because of these cultural habits, no-one can touch a newborn, worse if it’s a man. If women are clean and not hot [neither menstruating nor recent sexual relations] they can … We are stuck with this problem which has a large influence.”—International NGO respondent

#### Programme evaluation

The APE programme is still in the initial phase of implementation, and hence performance results are not yet available. Although the operational guide (Ministério da Saúde, 2011a) contains all the relevant tools for monitoring and evaluation, it is still not clear whether the reporting systems are effective.

“… we are still at the initial phase of implementation, and we have just introduced the logistics monitoring and evaluation tools now … we believe they will work properly because we will perform a good follow up and we will have partners’ support … I believe very much on this programme but we still have to improve it very much.”—Government respondent [3]

Although standardized registers for APE’s and reporting documents were approved, disparate donor/partner/NGO requirements have led to a multiplicity of monitoring and evaluation tools and reporting channels. This lack of standardization has seriously weakened the information system. Poor supervision likely compounded the problems, leading to inconsistencies and redundancies and serious under- and over-reporting. In an attempt to address the deficiencies, the National Institutes of Health and the National Directorate of Public Health in the MOH (Ministério da Saúde, 2012a) have recently developed key indicators to evaluate performance trends. Although the government and partners have great interest in the demonstrated impact of the APE programme, they have not invested in a research agenda to generate evidence of impact, or to more routinely gather feedback from community beneficiaries. Sporadic field assessments have, however, been undertaken.

#### Communications

Good relationships and communications, fundamental to strategic planning, are also key to ensuring sustainability in the implementation phase. Communication among stakeholders at national level through the Technical Working Group has been good, functioning well to coordinate and disseminate the programme.

But at other levels of the health system, communication has been poor. The lack of communication with districts meant that while the MOH emphasized that APEs should be based in their communities, with 80% of their activities focused on health promotion and disease prevention and only 20% on curative and first aid activities, some districts continued building health posts, thus emphasizing their curative roles.

“… while on the one hand the central policy decided that APE will not have a fixed post, the district was not warned and even today it continues to still wish to build health posts for APE, even with the existing policy already defined … the district should have been involved during these [policy development] steps … ”—Multilateral organization respondent [7]

As stated earlier, lack of engagement with the Ministry of Finance has contributed to the government failure to pay stipends to APEs.

### External factors

#### Funding stability

Currently, the APE programme is completely dependent for implementation on external aid. The government has insisted that donors pay for the programme comprehensively, rather than support discrete inputs. External resources therefore fund the recruitment and training of APEs, their supply of medicines and equipment, payment of stipends, supervision and monitoring of activities.

“… I think that the ministry managed to do it involving from the beginning all key organizations … when some organizations started saying that they could only provide training … I will not give incentives, I can do the supervision, the ministry said we all will do things as we agreed since the beginning ….”—NGO respondent [15]

This reliance on external funding is not without its shortcomings. Although APEs have government contracts, the funds come from NGOs and donors who cannot establish long-term contracts. This creates a precarious situation for APEs and increases the risk of attrition: ‘many partners come with projects of 3–5 years and they cannot commit themselves for more’ Government respondent [14].

Apart from creating uncertainty for APEs, the reliance on short-term project-based funding also constrained and fragmented scaling up of the programme.

“… the stipend is being the major constraint … it does not allow for a rapid scale up is exactly because the ministry imposes assurance of availability of funds to pay this stipend before training starts … that is, if I want to train APE from a given district, I have to assert that I have the stipend, at least for some period … ”—Government respondent [18]

**Figure 1.**
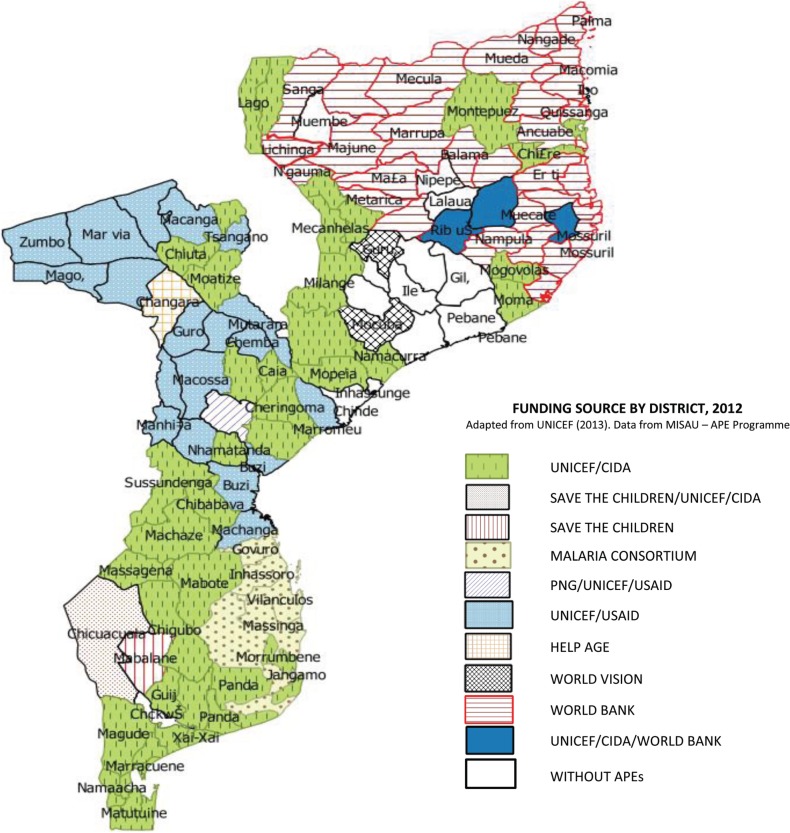
Heterogeneity and multiplicity of funding sources to the APE programme in Mozambique by districts, 2012.

As of June 2013, in the central province of Zambézia (the second most populous province with over 4.5 million people), the ratio of APEs to the population was 1:46 000. In Inhambane province further south the ratio was 1:5000. This is partly due to the segmented and heterogeneous nature of donor funding (Figure 1). In 2012, external resources constituted 56% of total health expenditure in Mozambique (WHO, 2014). As well as often being limited to a geographical area, each partner has its own agenda that may not be fully synchronized with national policy.

“NGO’s financing … goes only to a few provinces and not the others, partners who can finance this and that … we need to find a platform that is formal.”—Multilateral organization [21]

In addition to donor dependency, estimated total spending on health accounted for 6.2% of GDP in 2009, or only US$27 per capita, well below regional averages. Recently donors have delayed disbursements pending the MOH response to the 2011 Global Fund audit recommendations (Ministério da Saúde, 2012b, 2014a). This decrease of external support combined with a decrease in government funding for health may also hamper the government’s ability to invest resources in the APE programme (WHO, 2014).

#### Political support

As a pioneer of primary health care, the government has provided strong political support to the APE programme since 1978 (Lindelow *et al.*, 2004). In 2004, the government renewed its commitment to community health through a series of national consultations and policy documents (Ministério da Saúde, 2004, 2007b). More recently the government’s commitment to achieving the MDGs, and in particular to reducing child mortality, supports the APE programme, including iCCM.

Although the MOH has shown strong leadership and coordinated the programme, some interviewees suggested that to trigger more political involvement of community leaders and district authorities, the Prime Minister should have been invited to launch the programme.

“… it would have been very important to have the Prime Minister come forward and say this is the way we are going and there is no way back. We have missed that. And so that political priority is still a challenge because I’m not 100% clear on what is going to be happening later on.”—Multilateral organization respondent [21]

#### Partnerships

The government forged strong relationships with NGO partners working with communities to implement the programme. Some of these partners pressed strongly for implementation as they had made commitments to donors to meet defined targets for iCCM.

“… the alliance of organizations … Malaria Consortium, UNICEF and Save the Children. … These three organizations had budgeted schedules and could not lose money, they allied themselves to pressure the Ministry to fulfil the programme so that they could also meet the targets set in their plan.”—Multilateral organization respondent [2]

As mentioned earlier, community level actors were not extensively engaged or mobilized in the planning process, although they were more involved in implementation, beginning with choosing their APEs. A joint field assessment found that communities were satisfied, as they benefited from the new APEs, who were considered to be performing well (Ministério da Saúde, 2012b).

“… the few numbers that we have as feedback are impressive, are positive … , so I see at ground level more enthusiasm, a reality that is often completely different from what happens at the level of policies …”—Multilateral organization respondent [5]

Although this support was taken as a positive sign by policy makers, no strategy is in place to further mobilize communities or channel their support to strengthen the sustainability of the APE programme.

#### Public health impact

Although evidence on public health impact from other countries was key to acceptance of iCCM by both government and partners, there is as yet no evidence of public health impact in Mozambique. The expected public health impact of the programme can, if communicated effectively, lead to further support from communities, government and partners.

“… technically there is a lot of work, I really hope the programme will start generating some data soon, through which we can witness and count the gains … ”—Multilateral organization respondent [5]

As mentioned earlier, although both government and partners are keen for the APE programme to demonstrate impact, a strategy to generate such evidence in Mozambique has yet to be elaborated or supported.

## Discussion

Based on the framework proposed by Schell *et al.* (2013), this article analysed facilitating and constraining elements to sustainability with regards to the revitalized APE programme in which iCCM is embedded. Although iCCM was integrated into an NHS, serving communities distant from health centres, numerous policy and system challenges inhibit its likely sustainability. Successful technical coordination in policy development resulted in the MOH improving on past configurations of the APE programme. The MOH made strategic decisions to pay APEs, authorize their prescribing abilities, foster guidance, support operational planning and incorporate previously excluded ‘old’ APEs. These measures demonstrate strong government commitment to the APE programme from policy design to programme adaptations based on implementation experience.

Notwithstanding these important efforts, policy negotiations were focused in Maputo and at central levels without inclusion of other key stakeholders, such as the Ministry of Finance, district health officials, APEs themselves or beneficiary communities. Uncertainty remains with regards to the integration of APEs into the civil service and their long-term retention, given their short-term contracts and low compensation vis-à-vis market rates. Reliance on NGOs and donor funding has led to geographic distortions in implementation and limitations in scaling up, alongside struggles in harmonizing and strengthening domains that are notoriously weak in many health systems: supervision, supply chain management, monitoring and evaluation. Of particular concern, is the extent of reliance on external funding at a time when both external and government funding are declining.

Although we sought to understand the policy processes behind the relaunching of the APE programme to assess its sustainability and that of iCCM embedded within it, our work is not without limitations. Data collection took place over a brief period of time and many respondents were not pursued if they did not respond to multiple efforts to recruit them. High-level policy makers had limited time and were not always amenable to being interviewed alone, increasing the likelihood of giving official ‘party-line’ responses. Some respondents could not remember key events or processes accurately or had moved on to other positions since the relaunching of the programme. Despite these limitations, the study is led by national researchers with long-term engagement with community health and a deep understanding of national history and context. Efforts to interview respondents from various stakeholder positions, triangulation with the document review, validation meetings where preliminary findings were shared with key stakeholders and internal study team debriefings during data collection and throughout analysis helped to improve the quality of the study.

Many challenges encountered with iCCM and APEs in Mozambique are quite similar to the experience of implementing iCCM in Malawi and include weak supply chain systems, lack of a career path for CHWs and the associated risks of retention and motivation, weak supportive supervision and multiplicity of the reporting requirements imposed by donors/NGOs (Nsona *et al.*, 2012; Callaghan-Koru *et al.*, 2013). In contrast, Iran provides an example of a sustainable CHW programme embedded in an NHS where the CHWs are full-time employees of the government health service with refinements made to increase their educational level and add more tasks (Javanparast *et al.*, 2011). More policy-oriented analysis of scaling up iCCM in Nicaragua (George *et al.*, 2010) and of CHWs in Zambia (Zulu *et al.*, 2013) show that while addressing these technical considerations are important, considerable negotiation and political skill are required in fostering such initiatives including, seeking buy-in from multiple levels of government and other stakeholders such as professional bodies and unions, using monitoring data strategically and eliciting support from communities and health workers alike.

Although we have represented Schell *et al.*’s (2013) framework categorizing organizational and contextual domains essential for supporting programme capacity for sustainability in a tabular format, the framework in its original form lists the domains as a series of interacting and closely relating spheres resembling a compass. It has strategic planning at the centre, and funding stability, partnerships, programme adaptation and communications as its main north, south, west and east axis. Although all domains may be important, such a visual representation coheres with our findings from Mozambique, which also suggest that certain domains were strategically more influential than others. Certain domains may also be more in the control of programme managers than others, and more easily fostered than others. Some domains may need further conceptual refinement. Although public health impact is valued by the framework, other types of evidence may be valued by policy makers for health systems decision making (Peters and Bennett, 2012). Although community partnerships are essential for community-based programmes, in the context of low- and middle-income countries, these also entail partnerships with local and international NGOs and other development partners. A key finding from our research is that it is at times hard to categorize each of these domains as entirely positive or negative, due to the many nuances involved.

In Mozambique, although strategic planning did play a crucial role in setting the direction of the APE programme, attempting to relaunch it on a stronger foundation than its predecessor, two priority issues echo with ramifications throughout the programme. The first is the role of APEs within the health system, with regards to their integration into the public sector workforce, their retention in the programme and corresponding community linkages. Although the revitalization did imply more professionalized APEs, the programme still values its community rootedness. APE programme stakeholders need to periodically review current and future plans for the services they expect APEs to deliver, because this will affect the CHW profile they will need and how they link up with other human resources for health and supportive community structures. The current unbalanced ratio of male to female APEs requires attention particularly considering the gendered dimensions of reaching key target groups, whether female caregivers of sick children or pregnant or postnatal women and newborns (George, 2008; Richards *et al.*, 2013).

Very closely linked to strategic decisions and adaptations related to the APE’s role in the health system is the manner in which the programme is funded. The reliance on NGO and donor funding, while representing important, also represents transactions costs in terms of harmonization and skewed and staggered implementation. Mandatory requirements to demonstrate funding accountability and short-term results often lead to donors promoting a state of fragmentation and redundancy (Cassels and Janovsky, 1998; Hutton, 2002). Donor and government spending on health is declining in Mozambique and general trends of donor funding crowding out government spending have been found in certain country contexts (Murray *et al.*, 2013). However, challenges in measuring and interpreting aid flows (Sridhar and Woods, 2010; Garg *et al.*, 2012) make it hard to assess whether such trends imply governments restricting spending due to distrust of unpredictable foreign funding flows or reallocating spending to other areas due to the substitutive effect of foreign aid (Ooms *et al.*, 2010).

Our analysis of APE programme sustainability, and therefore iCCM sustainability, does indicate that the government did make notable efforts in certain key domains that were within its control, whether in terms of strategic planning, organizational capacity or programme adaptations. Nonetheless, certain external factors such as funding stability had an overriding and at times counteracting influence over these efforts. A sustainability analysis such as this highlights key strengths and weaknesses, but also points to levers that have to date been relatively under-utilized by the APE programme. Future interventions and research may be able to tell if, e.g. strengthening partnerships and communications could generate positive synergies with regards to further political support and public health impact with positive effects on fostering funding stability.

## Concluding remarks

Our analysis represents a nuanced assessment of the various domains that influence APE programme sustainability, highlighting in particular the crucial role of strategic decisions around the role of the APE in Mozambique’s health systems and the negotiations regarding the financial foundation of the programme. These considerations are not dissimilar to those identified by CHW evaluations undertaken 25 years ago (Berman *et al.*, 1987; Gilson *et al.*, 1989), which found variable implementation and success due to ‘unrealistic expectations, poor initial planning, problems of sustainability and the difficulties of maintaining quality’ (Gilson *et al.*, 1989). To further support efforts to not repeat failures from the past, issues related to sustainability, particularly its organizational and contextual determinants, need to be brought back to the heart of APE programme strengthening and iCCM policy support.

## Ethics review

Ethical review was sought from the Mozambique National Bio-ethics Committee and the JHSPH IRB. The study was approved by the Mozambique review board and exempted by the JHSPH IRB.
